# Bronchial artery embolization for haemoptysis in patients with lymphoma and leukaemia

**DOI:** 10.1093/bjrcr/uaaf036

**Published:** 2025-07-17

**Authors:** Ruben Geevarghese, Elena N Petre, Etay Ziv, Ernesto Santos, Lee Rodriguez, Vlasios S Sotirchos, Ken Zhao, Stephen B Solomon, Erica S Alexander

**Affiliations:** Interventional Radiology Service, Department of Radiology, Memorial Sloan Kettering Cancer Center, New York, NY 10065, United States; Interventional Radiology Service, Department of Radiology, Memorial Sloan Kettering Cancer Center, New York, NY 10065, United States; Interventional Radiology Service, Department of Radiology, Memorial Sloan Kettering Cancer Center, New York, NY 10065, United States; Interventional Radiology Service, Department of Radiology, Memorial Sloan Kettering Cancer Center, New York, NY 10065, United States; Department of Research and Technology Management, Memorial Sloan Kettering Cancer Center, New York, NY 10065, United States; Interventional Radiology Service, Department of Radiology, Memorial Sloan Kettering Cancer Center, New York, NY 10065, United States; Interventional Radiology Service, Department of Radiology, Memorial Sloan Kettering Cancer Center, New York, NY 10065, United States; Interventional Radiology Service, Department of Radiology, Memorial Sloan Kettering Cancer Center, New York, NY 10065, United States; Interventional Radiology Service, Department of Radiology, Memorial Sloan Kettering Cancer Center, New York, NY 10065, United States

**Keywords:** bronchial artery embolization, lymphoma, leukaemia, haemoptysis

## Abstract

Haemoptysis in patients with lymphoma and leukaemia can present a therapeutic challenge, given that it is often associated with underlying impairments of haemostasis and immunosuppression. Bronchial artery embolization (BAE) is a mainstay in the treatment of haemoptysis, typically in those requiring emergent management. In this case series, the role of BAE in patients with lymphoma and leukaemia was evaluated. A total of 5 patients were identified between August 2010 and August 2022. Three patients were diagnosed with lymphoma (classical Hodgkin’s lymphoma, diffuse large B-cell lymphoma and extra-nodal marginal zone lymphoma) and 2 patients were diagnosed with leukaemia (1 patient with acute myeloid leukaemia and the other with chronic lymphocytic leukaemia). All patients were thrombocytopenic [77.6 ± 28.5 × 10^9^/L (mean ± SD)], at presentation. Three patients had concurrent lung infection at the time of their presentation. Technical success was achieved in 4/5 patients (80%). Clinical success was obtained in 4/5 patients (80%). Recurrence following embolization was seen in 2 patients. Three patients died within 30 days following embolization (from deteriorating respiratory function). Bronchial artery embolization for haemoptysis in patients with lymphoma and leukaemia is safe and feasible. Concurrent lung infection is potentially of significance with regard to initial presentation and overall outcomes following embolization. In select patients, BAE may provide a therapeutic option, though further investigation is required.

## Introduction

Bronchial artery embolization (BAE) has an established role in the management of haemoptysis, following its first description in 1974.^1^ The technique has since evolved with regard to its indications, technical approaches and therapeutic efficacy.^2–4^

Patients with lymphoma and leukaemia presenting with haemoptysis not uncommonly present a therapeutic challenge given the often associated underlying impairments of haemostasis.^5^^,^^6^ Additionally, due to inherent immune impairment,^7^ such patients may be more predisposed to infections and consequently develop infectious complications, which may manifest with haemoptysis. Medical measures such as treatment of potential underlying reversible causes can often take time to take effect and may have limited efficacy.

In this case series, we aimed to evaluate the safety and effectiveness of BAE in this patient population.

## Case series

### Informed consent statement

Due to the posthumous status of most included patients, it was not possible to obtain individual written consent. All data presented have, however, been sufficiently anonymized to prevent the identification of any 1 individual. Approval for this case series, including a waiver of informed consent, was also obtained from the institutional review board (IRB Protocol Number 16-402).

### Patient population

A single-centre retrospective review was undertaken of patients presenting to a large tertiary cancer hospital between August 2010 and August 2022. Patients were initially identified following a search of the picture archiving and communications system for patients undergoing BAE for haemoptysis. Additional inclusion criteria were: (1) patients with lymphoma and leukaemia. Exclusion criteria were: (1) non-lymphoma and leukaemia malignancies; (2) post-surgical/post-biopsy complications resulting in haemoptysis; (3) pulmonary artery embolization. Bronchial artery embolization was undertaken following clinical evaluation and discussion with the patient’s referring physician.

### Data collection

Relevant clinical information was reviewed from the patient’s electronic health record. This included prior and concurrent medical treatments, laboratory results, radiology and bronchoscopy reports, time to recurrence of haemoptysis symptoms and overall survival.

### Baseline patient characteristics

A total of 5 patients (3 female and 2 male) were identified. Median age was 55 years (range 46-69). Three patients were diagnosed with lymphoma (classical Hodgkin’s lymphoma, diffuse large B-cell lymphoma and extra-nodal marginal zone lymphoma), and 2 patients were diagnosed with leukaemia (1 patient with acute myeloid leukaemia and the other with chronic lymphocytic leukaemia). All patients were thrombocytopenic at baseline, 77.6 ± 28.5 × 10^9^/L (mean ± SD). Three patients had concurrent lung infection at the time of their presentation. Further baseline characteristics, past medical history and pre-procedural laboratory values are outlined in Tables 1 and 2, respectively.

**Table 1.** Baseline demographics.

Variable	
Number of patients	5
Median age (range), y	55 (46-69)
**Sex**	
Female	3 (60%)
Male	2 (40%)
Median body mass index (range)	31.4 (21.4-35.1)
**Previous anti-cancer treatment (%)**	
Chemotherapy	3 (60%)
Immunotherapy	2 (40%)
**Current anti-cancer treatment (%)**	
Chemotherapy	1 (20%)

**Table 2.** Baseline medical history and pre-embolisation work-up.

Clinical data	
Previous history of haemoptysis	3 (60%)
**Co-morbidity (%)**	
Systemic hypertension	1 (20%)
Diabetes	1 (20%)
Bronchiectasis	1 (20%)
**Concurrent lung infection (%)**	
COVID-19	1 (20%)
Unspecified pneumonia	2 (40%)
**Medication history (%)**	
Antiplatelet	0 (0%)
Anticoagulant	0 (0%)
**Substance use history (%)**	
Current smoker	1 (20%)
**Labs**	
Hemoglobin (g/dL), mean ± SD	8.32 ± 1.15
Platelets (×10^9^/L), mean ± SD	77.6 ± 28.5
INR, mean ± SD	1.21 ± 0.10
White blood cell count (×10^9^/L), mean ± SD	12.04 ± 9.11
Creatinine (mg/dL), mean ± SD	1.14 ± 0.61
**Imaging/direct visualization (%)**	
Contrast-enhanced CT	3 (60%)
Bronchoscopy	4 (80%)

### Definitions

High volume (massive) haemoptysis was defined as >100 mL/24 hours and low volume haemoptysis as <100 mL/24 hours. Technical success was defined as accessing the bronchial artery supplying the area of involved lung (identified on pre-procedure CT or based on bronchoscopy findings) with delivery of an embolic agent.

Clinical success was defined as a complete or partial reduction in the volume and/or frequency of haemoptysis in the first 24 hours following embolization. When there was either an increase in volume or frequency of haemoptysis, this was defined as recurrence.

### Embolization technique

Following common femoral artery access and introduction of a vascular access sheath (5-6 Fr), catheterization of the target parent vessel(s) was undertaken with a 5 Fr catheter (various catheters were utilized, dictated by patient anatomy and operator preference). When required, catheterization was then performed with a microcatheter (eg, Progreat, Terumo, Tokyo, Japan; Direxion, Boston Scientific, Natick, MA, USA). Embolic agents used included Embospheres (100-300 μm, 300-500 μm, and 500-700 μm—Merit Medical, South Jordan, Utah, USA).

### Adverse events

Intra-procedure and immediate post-procedure complications were recorded. Post-embolization adverse events in the 30 days following therapy were graded according to the Common Terminology Criteria for Adverse Events (CTCAE) v5.0.^8^ For completeness, this included adverse events that were not clearly attributable to the embolization procedure.

### Statistical analyses

Continuous variables are represented, where appropriate, as mean ± SD or median ± range. Categorical variables are represented as absolute numbers and percentages.

### Pre-procedural and intra-procedural findings

**Figure 1.**
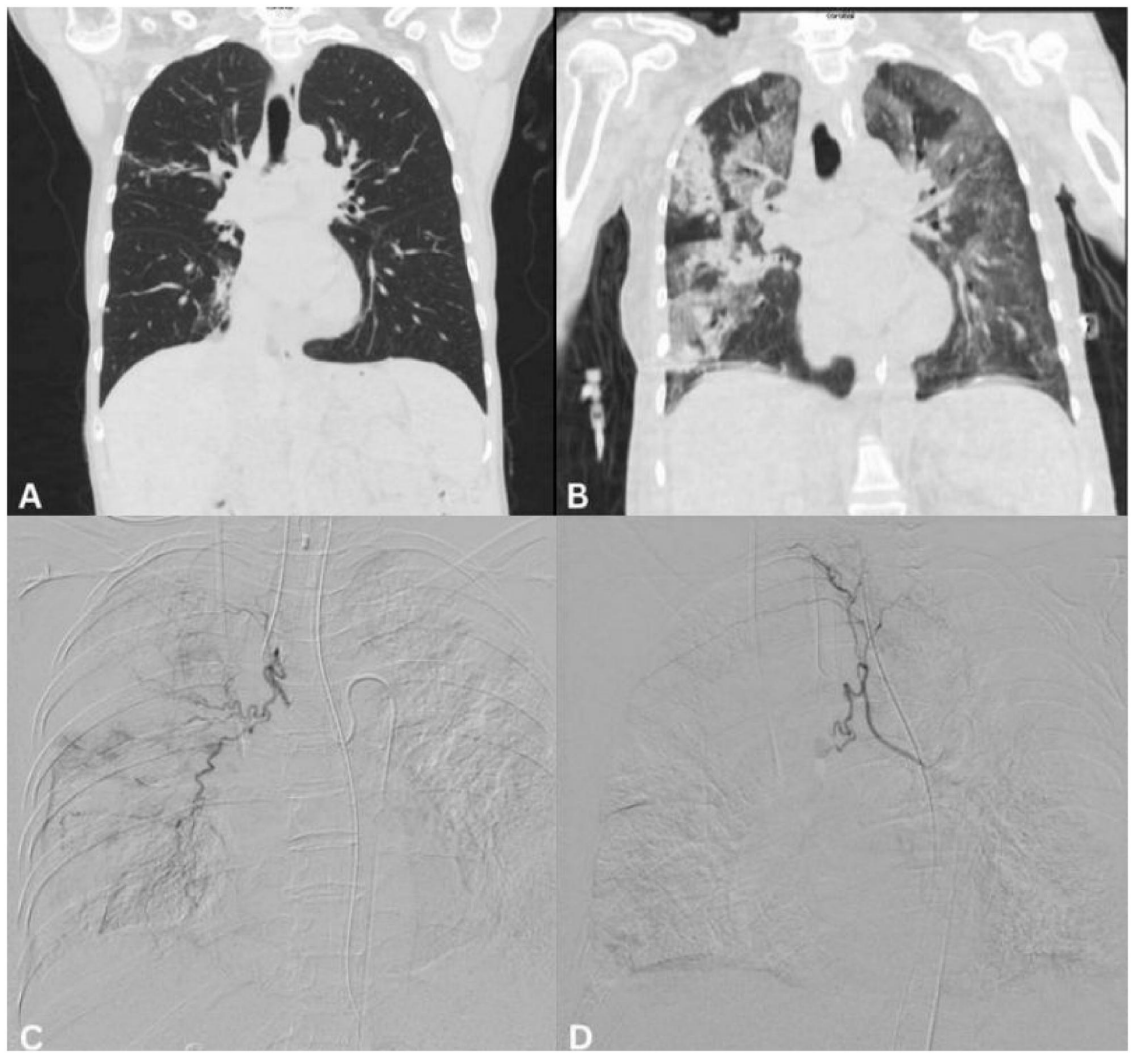
Embolization of haemoptysis in a leukaemia patient. 65-year-old female with chronic lymphocytic leukaemia. (A) Baseline coronal CT image demonstrates minor areas of atelectasis in the right upper and middle lobes (20 months before the date of embolization). (B) Coronal CT image, acquired during the admission for haemoptysis, demonstrates bilateral patchy ground-glass and consolidative opacities with a greater preponderance in the right lung. (C) Following bronchoscopy (which suggested evidence of bleeding from the right lung), selective angiography of the right bronchial artery demonstrated arterial hypertrophy and prominent parenchymal vascularity, correlating with CT and bronchoscopic findings. (D) The artery was then embolized to near-stasis with 100-300 and 300-500 micron Embospheres (Merit Medical, South Jordan, UT, USA), with significant reduction in parenchymal vascularity. Despite technical success and cessation of haemoptysis, the patient died 13 days later from progressive respiratory failure.

Two patients presented with large volume (massive haemoptysis) and 3 patients presented with small-volume haemoptysis. Pre-procedural contrast-enhanced CT was undertaken in 3/5 patients (66.7%) and bronchoscopy in 4/5 patients (66.7%). All patients underwent embolization under general anaesthesia. Arteriographic findings showed arterial hypertrophy in 4/5 (80%) patients. Technical failure in 1 patient (20%) was due to the bronchial arteries being too small to permit stable cannulation. The embolic agent in all cases was Embospheres (1 patient received 100-300 and 300-500 μm particles, 2 patients received 300-500 μm and 1 patient received 500-700 μm). An example case is outlined in Figure 1.

### Procedural success and post-procedure recovery/management

Median length of post-procedural hospitalisation was 15 days (range: 4-17 days). Technical success was achieved in 4/5 patients (80%). Clinical success following technically successful embolization was achieved in 4/4 (100%) patients. Two patients were extubated at the end of the embolization procedure. Recurrence was seen in 2 patients following embolization: in one patient at 2 days and in the other patient at 649 days.

### Overall survival and adverse events

Median length of follow-up was 16 days (13-2902 days). Median overall survival was 15.5 days (13-1586 days). Three patients died in the 30 days following embolization (all from respiratory failure—CTCAE Grade 5). Of the remaining 2 patients, 1 patient died from disease progression (at 1586 days) following embolization, with the final patient alive at last follow-up (2902 days following embolization). No evidence of non-target embolization (spinal cord ischemia or stroke) or access/procedure-related vascular complications were identified.

## Discussion

Bronchial artery embolization in patients with lymphoma and leukaemia is safe and feasible. Despite a high degree of technical and clinical success, the prognosis following presentation with haemoptysis in this patient population was poor.

Previous studies of transarterial embolization for malignant haemoptysis have predominantly included patients with primary and metastatic lung tumours; patients with lymphoma and leukaemia have typically been non-represented or composed a very small minority of patients, such as 2.2% in 1 paper of patients with lung tumours presenting with haemoptysis.^9^

In the current series, recurrence following technically successful embolization was seen in 50% of patients. This represents a higher incidence than a recently published meta-analysis of bronchial artery embolization for haemoptysis, reporting a rate of 23.7%; however, this analysis included patients with haemoptysis from multiple non-malignant etiologies.^10^ Studies focused on patients with haemoptysis and underlying neoplasm have reported higher recurrence rates ranging from 35% to 49%.^9^^,^^11^ Of note, despite recurrence (resolving spontaneously with conservative management), one patient in the current series benefited from 649 days of haemoptysis-free survival.

Overall survival in the current series was poor, with 60% of patients dying within 30 days of embolization. Previous studies in cancer patients presenting with haemoptysis have also demonstrated similarly poor survival rates.^9^^,^^11^ Additionally, development of haemoptysis was noted as an independent poor prognosticator for survival in 1 study of lung cancer patients,^12^ with a separate study identifying that patients presenting with large volume haemoptysis typically have a poorer outcome.^13^ Overall, this may raise questions regarding the utility of embolization; however, it is noteworthy that in 1 study comparing outcomes in patients who underwent embolization for haemoptysis to those who did not, survival was greater in the intervention group.^14^

Concurrent lung infection was noted in the majority of patients in the present series (3 patients—60%), adding a further dimension to the pathophysiology of the patient’s haemoptysis. It is possible that the patient’s underlying immunosuppression^7^ exacerbated the haemoptysis, particularly in the setting of their thrombocytopenia.

In the current series, there were no cases of non-target embolization. Despite all patients being thrombocytopenic, no access/procedure-related vascular complications were recorded. Overall, these are in keeping with low rates of serious complications in the published literature.^10^ One patient was unable to receive embolization due to the small size of their bronchial arteries. Vessel hypertrophy was, however, noted in 80% of patients in the current case series, but has more typically been reported in patients with chronic inflammatory or infective pathologies such as bronchiectasis and tuberculosis, respectively.^15^

There are several limitations to this case series. First, it represents patients over the course of 12 years with numerous operators undertaking the embolization procedure. Second, the sample size was small, limiting the generalizability of the study findings. Third, the severity of presenting haemoptysis (ie, volumetrically) likely played a pivotal role in the reported incidence of recurrence and survival.

Bronchial artery embolization for haemoptysis in patients with lymphoma and leukaemia is feasible and safe. Despite a high rate of technical success and initial clinical success, the overall rates of recurrence and survival in this patient population are poor. Concurrent lung infection and underlying immune status are potentially of significance with regard to initial presentation and overall outcomes following embolization. Future studies are needed to explore the relationships of these factors to clinical presentation and embolization success.
